# Use of the supercapsular percutaneously assisted total hip approach for femoral neck fractures: surgical technique and case series

**DOI:** 10.1186/s13018-016-0446-2

**Published:** 2016-10-12

**Authors:** Andrew W. Bodrogi, Robert Sciortino, David A. Fitch, Wade Gofton

**Affiliations:** 1 Division of Orthopaedic Surgery, University of Ottawa, The Ottawa Hospital, Civic Campus, 1053 Carling Avenue, Ottawa, ON K1Y 4E9 Canada; 2 Jones & Sciortino Orthopedics, 224 South Woods Mill Rd. Suite 255 South, 63017 Chesterfield, MO USA; 3 MicroPort Orthopedics Inc., 5677 Airline Rd., 38002 Arlington, TN USA

**Keywords:** Hemiarthroplasty, Femoral neck fractures, Minimally invasive surgical procedures, SuperPath, Supercapsular percutaneously assisted total hip, Bipolar

## Abstract

**Background:**

Femoral neck fractures are common injuries in the geriatric population associated with high morbidity and mortality rates. Studies have shown outcomes can be positively influenced by early postoperative mobilization. The supercapsular percutaneously assisted total hip (SuperPath) surgical technique has been shown to lead to early mobilization for osteoarthritic total hip replacement patients and as such has the potential to provide similar benefits in fracture patients. This manuscript provides a detailed description of this technique using hemiarthroplasty to treat femoral neck fractures and presents the first case series of this application.

**Methods:**

Seventeen patients with femoral neck fractures managed with this technique at two separate institutions were reviewed. In an attempt to minimize blood loss and enhance early mobilization, hemiarthroplasty utilizing the SuperPath technique was performed. The authors noticed decreased blood loss, operative time, and postoperative narcotic usage when compared to their previous experiences using traditional techniques.

**Conclusions:**

Early mobilization following femoral neck fractures has been shown to decrease mortality and morbidity. There is little existing literature on the use of tissue-sparing surgical techniques for this application, and none details the use of the SuperPath technique for it. The described case reports suggest the technique is a viable option for bipolar hemiarthroplasty to treat femoral neck fractures. Appropriately designed future studies are needed to confirm findings and definitively compare outcomes to traditional approaches.

## Background

Femoral neck fractures are common in the geriatric population and are associated with high mortality rates ranging from 11 to 36 % during the first year following fracture [1]. Previous studies have shown early postoperative mobilization can positively influence morbidity and reduce mortality rates in this population [2, 3].

In the last decade, minimally invasive surgical (MIS) and tissue-sparing approaches for total hip replacements (THR) have become popularized due to the potential for decreased muscular damage, decreased postoperative pain, decreased perioperative blood loss, and early mobilization [4–7]. It is plausible then that these techniques could provide the same benefits in the treatment of femoral neck fractures.

One tissue-sparing technique is the supercapsular percutaneously assisted total hip (SuperPath®) surgical technique (MicroPort Orthopedics Inc., Arlington, TN, USA). Similar to the SuperCap® (MicroPort Orthopedics Inc., Arlington, TN, USA) approach first described by Dr. Stephen Murphy in 2003, the SuperPath approach utilizes the interval between the gluteus medius and the piriformis to access the capsule and hip joint from a superior aspect and prepare the femur without releasing any muscles [8]. The femoral canal can then be accessed and broached directly through the femoral neck without dislocating the hip. By removing the need for hip dislocation, trauma to the posterior soft tissue structures is minimized and the posterior restraints to dislocation are preserved. The SuperPath approach also utilizes the percutaneous reaming portal of the PATH® (MicroPort Orthopedics Inc., Arlington, TN, USA) approach, first described by Dr. Brad Penenberg in 2004, to facilitate acetabular preparation from the normal trajectory without the release of the iliotibial band or short external rotators [9]. The result is a highly stable THR with good postoperative pain control and early restoration of function.

Early results for the SuperPath technique from the design surgeon, Dr. James Chow, have shown a mean hospital stay of 1.7 days with a low complication rate and radiographic results comparable to standard approaches [10]. In this single-surgeon series, there were no incidences of instability, neurovascular injuries, deep vein thrombosis, or infections. A recent multicenter study of nearly 500 primary THRs confirmed the results of the design surgeon cohort, reporting a mean length of stay of 1.6 days, a similarly low complication rate, and 91.5 % of patients being discharged routinely home [11]. While this surgical technique has been shown to benefit the typically younger primary THR patient, it may be of even greater value to the more fragile elderly femoral neck fracture population in that it has the potential to allow for an early return to pre-injury function while reducing the length of their unexpected hospital admission.

This manuscript presents an overview of this tissue-sparing technique when used for bipolar hemiarthroplasty to treat femoral neck fractures. The first cases detailing this application of the technique are also presented.

## Methods

All consecutive hip fractures treated using the SuperPath surgical technique at two centers between May 2013 and October 2015 were retrospectively reviewed. These cases were performed by single surgeon at each institution and represent their initial experience using the technique for treating fractures. All patients were treated with a hemiarthroplasty feature the PROFEMUR® Z or PROFEMUR® Gladiator uncemented femoral stems and the Gladiator® Bipolar Hip System (MicroPort Orthopedics Inc., Arlington, TN, USA). This review was granted approval by the institutional review boards at both institutions.

Institution A is a teaching institution where the majority of femoral neck fractures are managed through a lateral hardinge approach for patient care and educational purposes. Patients selected for the soft-tissue preserving SuperPath approach were patients where anticoagulation could not be reversed and/or monopolar electrocautery could not be utilized (i.e., pacemaker). Institution B is a not-for-profit hospital, and the surgeon performs the SuperPath technique on all hip fracture patients.

The hospital database was reviewed for patient age, length of stay (LOS), readmissions within 30 days, discharge status, transfusion, and any complications. LOS was defined as the number of nights the patient stayed in the hospital. Any readmissions that occurred within the first 30 days for any indication, not just those attributable to the procedure, were included. Discharge status indicated the disposition of the patient and was categorized as home, skilled nursing facility, or rehabilitation facility.

### Surgical technique

**Fig. 1:**
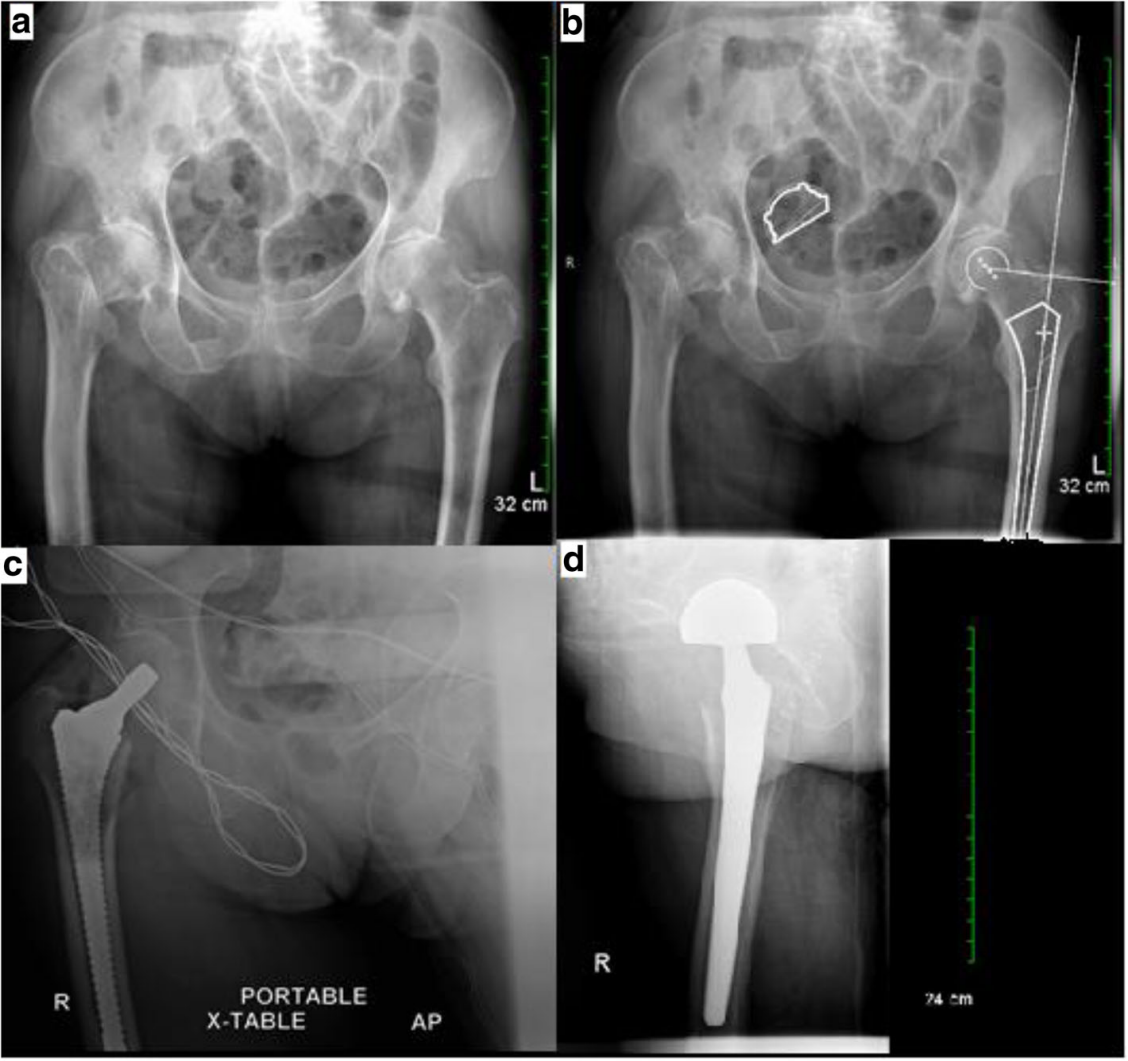
**a** Right low-energy subcapital hip fracture. **b** Computer software with preoperative template of the uninjured contralateral limb used to ensure restoration of postoperative offset and leg length. **c** Intraoperative film demonstrating appropriate broach depth, fit, and alignment. **d** Postoperative film lateral film

Prior to surgery, templating of the non-fractured contralateral hip is performed to identify the anticipated component sizes and the depth of insertion relative to the tip of the greater trochanter (Fig. 1). The computer system used to template cases was designed to template for THR, and therefore, there were no templates available specific to hemiarthroplasty. If preoperative templating is not possible, templating is performed on the fractured hip itself. This is less than ideal, as it is much easier to determine leg length and fit and fill with a non-fractured contralateral hip. The patient is positioned in the lateral position with the hip in 45° flexion and 10°–15° of internal rotation in high flexion (70°) to bring the longitudinal axis of the femur in-line with the direction of the gluteus maximus and tension the piriformis, similar to images provided in previous descriptions of the technique used for primary THR [12, 13]. Minimizing padding between the legs and placing the patient anteriorly on the table allows for more adduction to accommodate placement of the bipolar head. The foot is placed on a padded mayo stand to induce internal rotation and make the piriformis more easily palpable.

The incision is made in-line with the femur proximal to the greater trochanter, and the gluteus maximus is bluntly split. The author uses a headlight to improve visualization when performing this approach. A Zelpi retractor is placed, and the piriformis is visualized. A branch of the medial femoral circumflex artery generally lies over the tendon and can be cauterized. Using the interval between the gluteus medius and the piriformis, a blunt bent Hohmann retractor is placed under the gluteus medius over the anterior lip of the acetabulum. Lifting the knee slightly reduces the tension on the piriformis making it easier to pass a second retractor beneath the piriformis to protect it through the remainder of the case.

The capsule is then opened from the piriformis fossa along the line of the femoral neck and over the acetabular lip. Further elevation of the capsular flaps anteriorly and posteriorly allows for greater exposure. The blunt Hohmanns are then moved intra-articular around the femoral neck. A Romanelli retractor is placed to retract the capsule and maintain exposure. During primary THRs, care is taken to cauterize branches of the medial femoral circumflex artery close to the canal and broach start points. Failure to do so can lead to notable blood loss. However, in the setting of a femoral neck fracture, the authors have found that there is often minimal bleeding that they believe is secondary to kinking of the vessels and thrombosis.

The fracture can then be visualized, but the head is usually impacted on and often not in-line with the neck. The intact neck, not the relative position of the fractured head, is used as a guide to appropriate version. The starting point for the opening reamer is just anterior to the piriformis fossa, and the canal feeler should be used to ensure position within the canal. The round calcar punch is used to “canoe” out the femoral neck and head in order to insert the femoral broaches. A reverse curette is then used to identify the medial calcar or reduce any trochanteric overhang to allow appropriate broach placement. The appropriate broach is placed, and depth relative to the tip of the greater trochanter is compared to the preoperative plan. Soft tissue tension usually allows for broaching despite the femoral neck fracture, but if further stability is required, this can be achieved by an assistant stabilizing the knee during broaching.

The definitive neck cut is made using the broach as a guide. A Schanz pin in the head allows it to be removed and sized for the bipolar component. Following an assessment of the health of the acetabular cartilage, the trial bipolar head is placed in the acetabulum. The assistant pushes the knee towards the surgeon, delivering the femur to the surgeon. The trial neck is inserted (if a monoblock stem is planned, the trial neck must match the available monoblock stems; if a modular neck is planned, the preferred modular neck trial can be used). The surgeon has the option of placing the trial inner head on the stem and reducing into the outer bipolar head or reducing the trunnion into the preassembled bipolar component. The authors suggest the surgeon trial in the same way they plan to do the final assembly to ensure they have sufficient space to achieve the final reduction. Reduction is then achieved through a combination of hip abduction and internal rotation.

Once stability is confirmed, an intraoperative radiograph can be used to confirm restoration of leg length, offset, and appropriate canal fill. The trial modular neck is disassociated from the stem, and components are removed. The femoral head is inserted into the bipolar head and placed in the acetabulum. The stem is then inserted. As with any osteoporotic case, care must be taken with impaction to avoid the risk of calcar fracture. The reduction move is repeated and stability confirmed. Local intra-articular injection can be used for perioperative pain control, and the capsule is closed. The fascia and incision are then closed in the usual fashion.

## Results

There were 17 cases performed during the period of interest. Two patients were managed in institution A and 15 in institution B. The mean patient age was 80 years (range, 60–93). There were no instances of deep vein thrombosis or pulmonary embolism, and four patients (23.5 %) required transfusions. The majority of patients were discharged to a skilled nursing facility (66.7 %) after a mean LOS of 4.1 days (range, 3–9). The remaining patients were discharged home (6.7 %) or to rehabilitation (26.6 %). There was a single readmission within 30 days for a traumatic fall-related femoral fracture at 2 weeks discharge. There were no deaths or revisions for any reason reported.

## Discussion

This manuscript describes the successful use of a tissue-sparing surgical technique for bipolar hemiarthroplasty to manage subcapital hip fractures. While tissue-sparing and MIS approaches have the potential to result in early mobility and in turn reduced mortality and morbidity for the femoral neck fracture population, there have been relatively few published descriptions of their use in this application. A literature review returned only four prospective, randomized trials comparing MIS approaches to standard approaches for bipolar hemiarthroplasties in the treatment of femoral neck fractures.

Auffrath et al. found longer intraoperative times and increased pain associated with a modified Smith-Petersen approach with no difference in mobility as measured by the Harris Hip Score (HHS) at 6 months [14]. In contrast, Renken et al. noted significantly decreased pain and improved early mobilization with a direct anterior approach [15]. Roy et al. found no significant difference in all outcomes with a mini-incision posterior approach [16], while Kaneko et al. described a shorter “full weightbearing term” with their mini-incision posterior approach which utilized personally developed retractors [17]. As can be seen, the results of MIS procedures for hemiarthroplasties have been mixed. The currently described tissue-sparing technique may have a benefit over these techniques in that it does not require the cutting of muscles or the forcible dislocation of the femoral head.

In the described case series, the SuperPath approach was adapted to perform bipolar hemiarthroplasties to treat femoral neck fractures. The authors found this technique to be particularly useful in elderly patients with multiple comorbidities. In these cases, likely secondary to its muscle-sparing approach, there was an anecdotal noticeable decrease in postoperative pain and the subsequent need for analgesia compared to that seen in our typical fracture patients. From a decreased use of narcotics, there follows a reduction in the expected rates of postoperative delirium with its associated complications and extended hospital stays. Delirium is quite common following hip fracture surgery, with rates reported as high as 18–34 % [18–20]. One patient did experience postoperative delirium, but it was multifactorial in cause and likely associated with existing neurological comorbidities.

In the experience of the authors, this approach results in decreased blood loss as compared to the conventional direct lateral approach for use in treatment of femoral neck fractures and primary THRs. This agrees with the previously described multicenter study reporting a combined transfusion rate of just 3.3 % for nearly 500 primary SuperPath THRs [11]. An approach with minimal blood loss was ideal in preventing possible complications including cardiac events, strokes, and the need for perioperative blood transfusions.

Another potential benefit to the patient is the theoretical decreased risk of posterior dislocation. The described technique allows for the preservation of the short external rotators and avoidance of atypical positioning of the femur which may stretch local soft tissues that could increase the risk of dislocation. The randomized prospective MIS posterior approach studies returned in the literature search did not show this benefit, but those approaches also sacrificed the external rotators [16, 17]. A recent retrospective analysis of a modified posterior approach that preserved the external rotators reported significantly lower dislocation rates versus a standard posterior approach [21].

Aside from the benefits to the patient, the use of the described surgical technique also provides potential advantages to the surgeon. There are essentially no restrictions on the implant design that can be used and no need for special tables or equipment aside from the supplied instrumentation, as are sometimes required by other techniques. The technique utilizes an approach that is familiar to orthopedic surgeons as it is equivalent to the approach for a femoral nail. It can also be easily extended to a classic Kocher-Langenbeck incision should the surgeon have concern or run into complication.

Another benefit to the surgeon is that it is possible to straightforwardly change from a bipolar hemiarthroplasty to a THR during surgery using the same soft-tissue sparing window. In the author’s experience using this technique for THRs in the osteoporotic population, the femoral side has not presented significant difficulties. The acetabular side though, which must be accounted for if the surgeon switches to a THR, has presented the greater challenge in that osteoporosis can result in an increased risk of eccentric ream and acetabular wall or column fracture. If there is concern with the ream or placement of the acetabular component, the incision can easily be extended to a traditional Kocher-Langenbeck approach.

One potential risk of using this technique in this application is that of a periprosthetic fracture. In the described patient population, with high rates of osteoporosis, there is always the inherent increased risk of periprosthetic fracture with stem implantation. There is evidence to suggest that these rates may be elevated in MIS approaches in both the arthritic [22, 23] and osteoporotic populations [10, 17, 24, 25], likely related to the decreased visibility. By broaching with the neck/calcar in place using the described technique, one may be relatively protected from this complication when compared to other techniques due to the theoretical reduction in hoop stresses. In the osteoporotic population, neck fractures are often high, so the residual neck may be somewhat protective. However, at the time of definitive stem impaction after the neck has been resected, this protective effect is lost. Nonetheless, the authors do not feel that this risk is any greater with the SuperPath approach in comparison to a more open approach.

The authors experienced one intraoperative periprosthetic fracture when using this approach for placement of a bipolar hemiarthroplasty. In this case, the incision was slightly extended, a portion of the iliotibial band was split, and the piriformis and obturator internus muscles were detached to allow for the assessment of fracture extension and the placement of a cerclage wire. Intra-operative visualization as well as intraoperative and formal postoperative radiographs demonstrated no sign of extension beyond that observed in the operating room. Hence, the patient was allowed to weight-bear postoperatively and recovered without incidence. The authors have also seen a partial greater trochanteric fracture secondary to the pull of the piriformis muscle when using the technique for THR. This can be avoided by releasing the piriformis if overly tight. In this case, there was a small avulsion fracture and the peri-fracture soft tissue remained intact as a sleeve. No further management was required, and it healed without an alteration in postoperative activity.

Another topic of interest is that the included case studies use uncemented femoral stems. In 2014, the National Institute for Health and Care Excellence (NICE) in the UK issued a recommendation to use cemented femoral stems in the treatment of femoral neck fractures [26]. Based upon these guidelines and other publications, it is often accepted that cemented femoral stems should be used in the elderly population. However, the use of cemented stems does have some drawbacks including more difficult revision surgeries, potentially longer surgeries, and cardiac complications. A recently published randomized controlled trial [27] and meta-analysis [28] have shown no difference in outcomes with either cemented or uncemented stems, suggesting it may be possible to exploit the theoretical benefits of uncemented stems without exposing patients to additional risks (e.g., future fractures) [29]. In the current study, there was a single fracture due to a fall. Further follow-up of these patients and additional studies are needed to further evaluate the use of uncemented stems for elderly patients.

There are several limitations to the current study. The first is that the study is a retrospective case series. Second, the study only has follow up through the first 30 days following surgery. While this immediately postoperative period is general when the greatest benefit to fracture patients would be seen in the reduction of LOS, complications, and mortality, it is also possible that such a short follow-up time could result in the missing of complications or information for patients treated at other hospitals. Finally, the use LOS as an endpoint should be interpreted with caution. In some cases, extended LOS might be better for the treatment of older patients.

## Conclusions

In conclusion, the described case series suggest the adaption of the SuperPath technique for bipolar hemiarthroplasty is a viable option to treat femoral neck fractures in the geriatric osteoporotic population. Due to its minimal dissection and lack of hip dislocation, it carries the observed benefits of reduced blood loss, decreased postoperative pain, and possibly quicker return to function. Appropriately designed future studies are needed to confirm these findings and definitively compare outcomes to traditional approaches.
